# Commentary: Sexual Dimorphism of Facial Width-to-Height Ratio in Human Skulls and Faces: A Meta-Analytical Approach

**DOI:** 10.3389/fendo.2018.00227

**Published:** 2018-05-07

**Authors:** Martin G. Köllner, Kevin T. Janson, Oliver C. Schultheiss

**Affiliations:** Human Motivation and Affective Neuroscience Lab, Department of Psychology, Institute of Psychology, Friedrich-Alexander University Erlangen-Nürnberg (FAU), Erlangen, Germany

**Keywords:** anthropometry, facial width-to-height ratio, meta-analysis, organizing hormone effects, puberty, sexual dimorphism

The facial width-to-height-ratio (fWHR), the bizygomatic width divided by upper face height [prosthion-to-nasion distance; e.g., Ref. (1)], is a morphometric index in humans. Meta-analyses demonstrate that fWHR is linked to aggression in men (2) and dominance behavior across both sexes (3). fWHR is considered a marker of the effects of pubertal testosterone (4) and perhaps other steroid hormones [see discussion in Ref. (5)] on the developing brain which in turn affect adult behavior or personality. fWHR is often considered sex dimorphic, with men having higher ratios than women [e.g., Ref. (6); see meta-analysis by Geniole et al. (3)].

We are grateful to Kramer (7) for conducting a comprehensive and carefully done meta-analysis that calls into question fWHR’s status as a sexually dimorphic feature. Nevertheless, it would be premature to use his results to dismiss fWHR as a valid marker of hormone exposure for two reasons: first, the conclusions of the meta-analysis regarding fWHR’s lacking sexual dimorphism suffer from some inherent flaws. Second, there are better and more structured ways to approach the valuable body of data already generated by this line of research.

First and foremost, Kramer’s meta-analysis delivers not a falsification, but a skull-based replication of the overall sexual dimorphism found in the previous meta-analysis by Geniole et al. (3), as Kramer (7) (p. 417) briefly mentions (*p* = 0.02, or 0.002 after outlier exclusion). In addition, the conclusion that the sex dimorphism observed by Geniole et al. (3) vanishes when examining only face-surface-based fWHR studies is drawn based on a non-significant moderation analysis (*p* = 0.92) and despite a lingering trend-level difference (*p* = 0.07), with the confidence interval barely including 0 [−0.01, 0.25]. In fact, there is no real change in observed effect sizes after excluding the skull samples (8 of 32 samples)—*d* even slightly increases from 0.11 to 0.12! Similarly, Kramer claims that white faces do not show a sexual dimorphism in fWHR, but he obtains this result only after dividing the Geniole et al. sample along the non-significant moderator source (skulls versus faces) and ethnicity (non-significant in Kramer’s own study sample, *p* = 0.19). Thus, this finding, only based on the remaining quarter of all samples (*k* = 9), is not convincing. Moreover, Kramer’s analytical strategy—drawing conclusions based on non-significant moderators as if they were significant—is at odds with his previously described dismissal of a lingering trend-level sex difference as non-significant and even reporting this dismissal in the abstract. To conclude, Kramer’s meta-analysis may provide a cautionary note regarding sexual-dimorphism claims for fWHR, but surely does not falsify them.

Finally, over-focusing on skull-based differences like in Kramer’s literature search is inappropriate: it might not be necessary that proposed markers show up on the skull, just because Weston et al. (8) discovered fWHR this way. Soft tissues, too, might exhibit sexual dimorphisms [as Kramer (7) (p. 415) acknowledges] and thus index exposure to organizing hormone effects. It suffices to show that there are morphological differences that are sex dimorphic, related to pubertal steroid hormones, and predictive of behavior.

Nevertheless, Kramer’s meta-analysis (7) calls attention to an important issue: fWHR may not be as promising an indicator of hormone exposure as initially assumed, especially considering lacking associations between fWHR and polymorphisms in the androgen receptor gene or hormonal parameters (9). If a (seemingly) well-established marker’s sexual dimorphism is actually rather small and thus perhaps not very sensitive to hormonal effects, then it would be more prudent to conduct a systematic search for stronger markers of organizing hormone effects, based on large samples and starting with an explorative approach without pre-specifying the facial features of interest [perhaps based on classifiers, see Ref. (10)]. It will also be important to show that markers identified in this manner relate to actual brain structure and function and thus provide valid indices of hormonal effects on the central nervous system.

The search for valid markers can be accomplished by using the morphometric data generated by fWHR [see Supplemental 2 in Ref. (3), for an overview] and other anthropometric research and reanalyzing studies on relationships to behavior and personality. Besides unpacking the information underlying compound measures such as fWHR, we should measure additional points, ratios, and distances [e.g., Ref. (11); cf. Figure 1; see Ref. (9), for suggestions regarding multivariate approaches and evolutionary selection designs] of the face or even use automatic pattern recognition to derive such features. Afterward, we can systematically determine which features (or which combinations of features) are reliably (1) sexually dimorphic and (2) predictive of relevant criteria.

**Figure 1:**
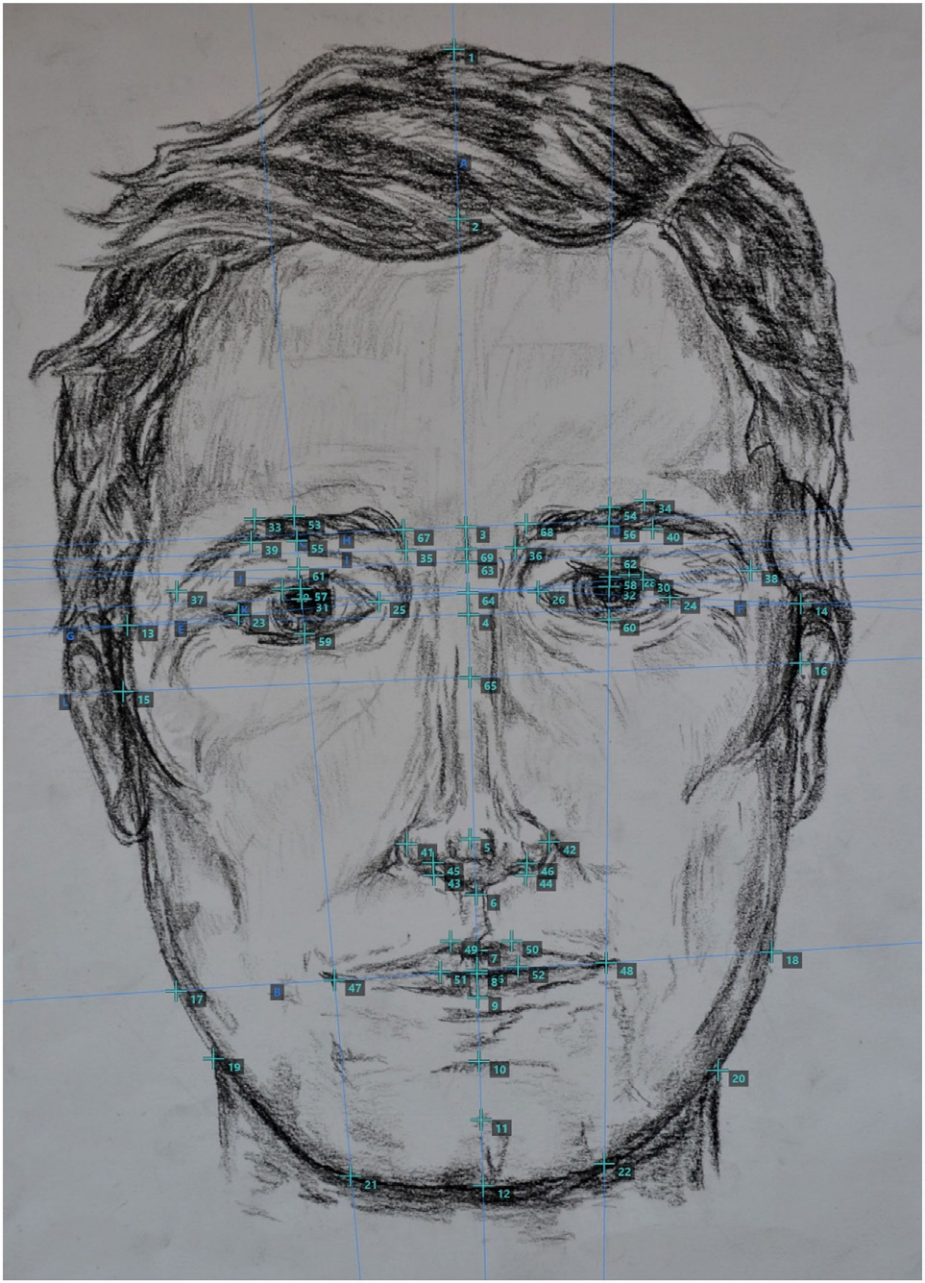
Sample drawing showing some examples of possible measuring points on the human face [measured *via* Face/Palm software (12)].

Moving further, the derived prediction models should then be validated in confirmatory pre-registered studies using 3D scans and the same behavioral or individual difference variables. Final validation—determining which marker candidates actually develop in a sex-dimorphic way—should come from longitudinal studies examining the same individuals before, during, and after the end of puberty and collecting hormone data as well as behavioral and personality data at each stage, respectively [see Ref. (13), for a promising design combining prenatal and pubertal testosterone assessment and a brain lateralization measure during task-engagement].

As an ultimate goal, the same approach should be applied to brain structure and function itself, *via* (f)MRI-studies, thus allowing a triangulation of body markers, brain features, and behavior. Until now, except for a few attempts in this direction [e.g., Ref. (14, 15)], there is a deplorable lack of such studies. If markers are truly indicative of organizing hormone effects on the brain that in turn contribute to behavioral development, then valid markers should be related to actual brain structure and function, which in turn should mediate the association between markers and behavior.

Resting on such a firm, data-driven basis, research on organizing hormone effects in humans will move away from over-focusing on the same small set of indicators that keep producing mixed results and toward making best use of the available data and achieving substantial progress.

## Author Contributions

MK wrote the commentary. KJ and OS provided edits and suggestions for revision.

## Conflict of Interest Statement

The authors declare that the research was conducted in the absence of any commercial or financial relationships that could be construed as a potential conflict of interest.
